# Pelvic Kidney: A Review of the Literature

**DOI:** 10.7759/cureus.2775

**Published:** 2018-06-09

**Authors:** Seif Eid, Joe Iwanaga, Marios Loukas, Rod J Oskouian, R. Shane Tubbs

**Affiliations:** 1 Department of Anatomical Sciences, St. George's University, St. George's, GRD; 2 Seattle Science Foundation, Seattle, USA; 3 Neurosurgery, Swedish Neuroscience Institute, Seattle, USA; 4 Neurosurgery, Seattle Science Foundation, Seattle, USA

**Keywords:** anatomy, anatomic variation, pelvic kidney, ectopic kidney, horseshoe kidney

## Abstract

Kidney development is a complex process that begins during the sixth to eighth weeks of life. Failure of ascent of the kidney will cause the kidney to remain in the pelvis i.e., pelvic kidney. Here, we review this entity in detail and illustrate such embryological derailment. In most cases, a pelvic kidney is an incidental finding and is usually asymptomatic. Anatomic variations of the renal vasculature have been reported in cases of pelvic kidneys and these are highlighted in this review. Clinicians who treat patients for renal or pelvic disease or interpret images of the pelvis should be well informed of the anatomy and embryology of the pelvic kidney.

## Introduction and background

**Figure 1:**
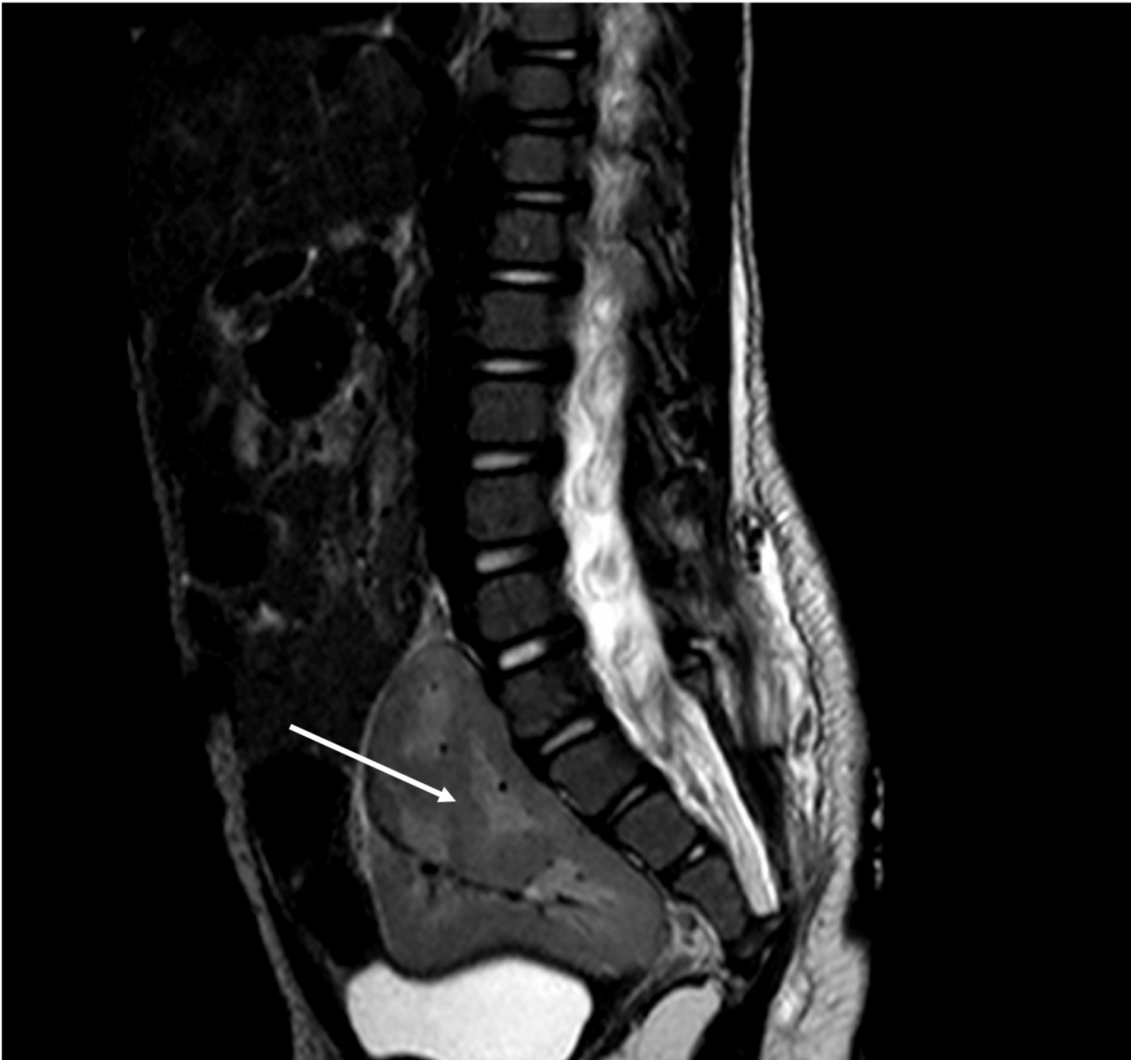
Sagittal T2-weighted magnetic resonance imaging (MRI) of the spine noting a pelvic kidney (arrow) This variant was found incidentally during the evaluation of a cutaneous flat capillary hemangioma, over the midline lumbar spine, to rule out occult spinal dysraphism. The patient had no history of pelvic pain or renal dysfunction.

**Figure 2:**
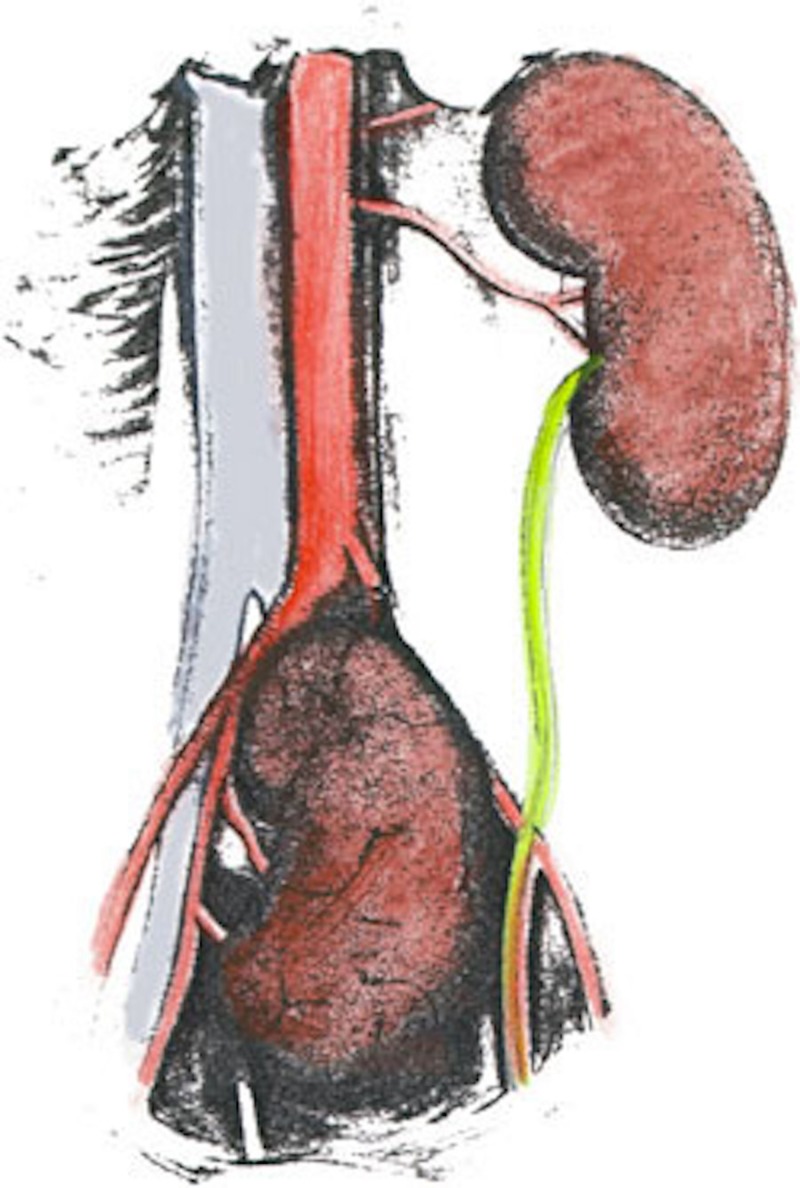
Drawing of a case of a pelvic kidney (modified from Brown M; 1894)

A pelvic kidney occurs when an error takes place during the ascent of the kidney in the early development stages, leading the kidney to remain in the pelvis, instead of the abdomen. The term that relates to the abnormal positioning of the kidney is called ectopic kidney. Different types of renal congenital anomalies exist; however, they are rare and usually asymptomatic. Despite the rarity, it is imperative to be aware of these anatomical variations during surgery and patient management. Here, we review this entity in detail and illustrate such embryological derailment (Figures 1-2).

## Review

Development of the renal system

The embryological development of the kidney is a complex process occurring between the sixth to eighth weeks of life, while the ascent of the kidney occurs during the ninth week. The developing kidney consists of three stages of development; these are the pronephros, metanephros, and mesonephros stages. The urinary system, as well as the reproductive system, are derived from the intermediate mesenchyme [1]. Although there is glomeruli development in the metanephros stage, it is ultimately the mesonephric kidney where the real maturation takes place. The metanephric kidney arises from the ureteric bud and invagination of the mesonephric duct [1]. The ureteric bud, which arises from the mesonephric duct, divides in a branching fashion to form the renal pelvis, calyces, and the collecting duct [2]. Initially, the kidney is located at the level of the sacral spine, it will then ascend while rotating along its axis to the level of the upper lumbar vertebrae [1-2].

Types of kidney anomalies in regard to shape and position

Due to the complex embryological development, there are different types of congenital anomalies that can occur such as the horseshoe kidney and the L-shaped kidney. Renal anomalies can be categorized into anomalies in number (renal agenesis), position (ectopic kidney), size (hypertrophy), and form (polycystic kidney) [3]. Fusion of the kidney can occur at variable locations; however, the horseshoe kidney is the most common fusion anomaly that leads to a pelvic kidney. Usually, fusion of the lower poles of the kidney by an isthmus gives rise to the typical presentation of the horseshoe kidney. Horseshoe kidneys are more common in men and are rare, occurring in only 1 of 400-600 people [4]. Another study revealed that only one case (0.4%) was found in 250 cadavers [5]. The characteristic U-shaped kidney will be prevented by the inferior mesenteric artery from ascent into the abdomen. An L-shaped kidney occurs when the lower pole of one kidney migrates and fuses with the contralateral kidney. Iwanaga et al. [6] reported one incidental case in a female cadaver during an anatomy dissection. In that case, these authors speculated that the right kidney was an ectopic kidney and that the L-shaped kidney was a result of fusing between ectopic and normal kidneys.

Variations in vasculature anatomy

The vasculature of the pelvic kidney has been reported as complicated and highly variable, which is due to the pelvic kidney retaining its fetal blood supply. Dretler et al. [7] reported many variations at autopsy where the arterial supply was either single, double, or triple and the origin varied between the bifurcation of the aorta, the iliac artery, and the hypogastric artery. One study even reported an absent right common iliac vein [6]. Knowledge of these anatomical variations is beneficial for patients undergoing abdominal surgery with pelvic kidneys. Horseshoe kidneys are usually smaller and sclerotic with variable positions along the lumbar spine [4].

Discussion

Although pelvic kidneys are usually asymptomatic and are found incidentally, they can be associated with other conditions such as nephrolithiasis, ureteropelvic junction obstruction, and extrarenal calices [8-9]. It has been hypothesized that the ureteropelvic junction obstruction is due to the high insertion of the ureters into the renal pelvis causing delayed emptying [5]. Knowledge of anatomy and the vasculature can be obtained by imaging in order to assist in the management and prevent complications. Arteriography can be very beneficial in identifying the variant arterial supply to prevent blood loss, while a nephrotomography can be helpful in viewing the parenchyma and the collecting system [7]. This is particularly important in the preoperative management for patients undergoing abdominal operations such as kidney transplantation. Several cases exist in the literature reporting equal efficacy between transplanted pelvic kidneys and normal kidneys. [10-11] A horseshoe kidney can be transplanted en bloc or divided for two recipients, which can significantly increase the donor pool for patients requiring renal transplantation [10].

The pelvic kidney, however, can be associated with other congenital anomalies that may require further attention. For example, Dretler et al. [7] have reported cases associated with vaginal atresia, imperforate hymen, hypoplastic uteri, and rudimentary fallopian tube and ovary. These anomalies can occur as the mesonephric duct (also known as the Wolffian duct); they give rise to the urinary and reproductive organs.

A thorough knowledge of both normal and variant abdominopelvic anatomy is important to the clinician treating patients with pathology of this region [12-17]. In addition, imaging can play a significant role in identifying the variant anatomy to assist in the management of pelvic disorders. 

## Conclusions

Pelvic kidney is a type of congenital anomaly that has variable presentation and abnormal anatomy. This review highlights the key aspects of the pelvic kidney in order to improve patient management. However, much is yet to be discovered due to the variability and complexity of this congenital anomaly.
